# Evaluation of Renoprotective Effects of Our Locally Grown Green Coffee Beans against Cisplatin-Induced Nephrotoxicity in Swiss Albino Mice

**DOI:** 10.1155/2021/2805068

**Published:** 2021-10-12

**Authors:** Bati Leta, Chala Kenenisa, Tesaka Wondimnew, Tariku Sime

**Affiliations:** Department of Biomedical Sciences, Faculty of Medical Sciences, Institute of Health Sciences, Jimma University, Jimma, Ethiopia

## Abstract

**Introduction:**

Nephrotoxicity is the most common and severe side effect of cisplatin. Cisplatin causes nephrotoxicity through free radical production and debilitating cellular antioxidant capacity. Coffee is a commonly consumed drink and its ingredients have antioxidant roles that could bring benefits to patients affected by nephrotoxicity. Thus, the present study aimed to investigate the renoprotective effects of our locally grown green coffee beans against cisplatin-induced nephrotoxicity in Swiss albino mice.

**Methods:**

The posttest only control group design was employed on a total of thirty male Swiss albino mice. The mice were divided into five groups: group I (normal control group) received distilled water; group II (negative control group) received distilled water; and groups III–V (treatment groups) received 100, 200, and 300 mg/kg BW/day of green coffee bean extract for 14 days, respectively. Nephrotoxicity was induced in groups II–V by a single intraperitoneal injection of cisplatin (7.5 mg/kg). All mice were sacrificed after 14 days and blood was drawn to evaluate kidney function tests (serum creatinine and serum blood urea nitrogen). Besides, body weight, relative kidney weight, and kidney histopathology were investigated.

**Result:**

Our results showed that treatment of cisplatin alone (group II mice) significantly increased serum creatinine, serum blood urea nitrogen, relative kidney weight, and pathological damage to the kidney with a decrease in final body weight. However, low-dose green coffee beans (group III), medium-dose green coffee beans (group IV), and high-dose green coffee beans (group V) mice showed a significant dose-dependent decrease in serum creatinine, serum blood urea nitrogen, and relative kidney weight. Furthermore, the dose-dependent treatment with green coffee bean extract prevented the decrease in body weight gain and pathological damage to the kidney in mice.

**Conclusion:**

Our locally grown green coffee beans brought a dose-dependent ameliorative effect and a promising preventive approach against cisplatin-induced kidney damage in mice.

## 1. Introduction

Kidney diseases are known to be public health problems affecting more than 750 million people worldwide. A wide range of therapeutic drugs and environmental pollutants often contribute to kidney disease. Drug-induced kidney damage is a root cause of several kidney diseases, such as AKI, CKD, and ESKD, which eventually results in an increased risk of CVD and increased healthcare costs [1, 2]. The kidney performs essential functions in humans by maintaining the composition of the blood and its PH, averting the accumulation of waste products, and preserving balanced electrolyte levels. Similarly, drugs, endogenous metabolites that are important for maintaining physiological homeostasis, exogenous and endogenous toxins, and nutrients are all eliminated by the kidney as a net effect of glomerular filtration, tubular secretion, renal metabolism, and reabsorption [3].

Kidney damage can be seen as a change in the function and structure of the kidney. The damage to the kidney is caused due to its physiological vulnerability. Frequently, kidney damage may progress to AKI or CKD [4]. The period of renal function deterioration is a baseline to make a distinction between AKI and CKD. AKI is defined as an abrupt change in renal function and is commonly defined by changes in BUN and serum creatinine for less than three months. CKD is defined as the structural and functional abnormalities of the kidney that last longer than three months [5].

Cisplatin is a platinum-based chemotherapy agent widely used to treat solid tumors such as ovarian cancer, cervical cancer, and head and neck cancer [6]. Cisplatin is rapidly distributed to tissues with high concentrations in the kidneys, liver, ovaries, uterus, and lungs and its principal excretion site is the kidneys. Bone marrow suppression, gastrointestinal toxicity, ototoxicity, neuropathy, and especially nephrotoxicity are the major and severe side effects of cisplatin [7]. Kidney damage is reported in roughly 25–35% of patients treated with only one dose of cisplatin [8]. Although the exact mechanism is not fully understood, the generation of ROS, the reduction of antioxidant enzymes, and the involvement of inflammatory mediators were associated with cisplatin-induced kidney damage [9].

Investigators have considered several strategies for the management of kidney damage caused by nephrotoxicity. While these strategies have shown promising effects in a variety of experimental models, they do not show consistent benefit or have proved successful when used therapeutically [1]. Current kidney damage therapy is therefore empirical and used selectively without understanding the underlying etiology. Providing natural products, such as medicinal plants, is considered the most effective way to mitigate the risks of different diseases with fewer side effects [10].

Medicinal plants are the central foundations in the traditional systems of medicine due to their effectiveness, low cost, and affordability [11]. Nowadays, plentiful quantities of medicinal herbs have been utilized for the therapy of kidney and kidney-related diseases. Several studies have been done to investigate the role of the herbal agent in cisplatin-induced nephrotoxicity [12–14]. Among them, *Ginkgo biloba* [15], *Morus alba* [16], *Rubia cordifolia* [17], *Curcuma comosa* [18], pomegranate flower [19], *Nigella sativa* [20], grape seed [21], and ginger extract [22] are famous and studied for their beneficial role in cisplatin-induced nephrotoxicity.

Most of these herbal agents are rich in phenolic components such as flavonoids and anthraquinones that show potent antioxidant effects. They show a protective effect against cisplatin-induced nephrotoxicity through scavenging free radicals, downregulating oxidative stress proteins, preventing loss of antioxidant enzymes, and anti-inflammatory effects, hence decreasing BUN, creatinine, kidney tissue damage, apoptosis, inflammatory and oxidative stress [17, 18, 21, 22].

Coffee, which represents the fruit of the coffee plant, belongs to the Rubiaceae family. Coffee has two chief species: *Coffea arabica* and *Coffea robusta* [23]. Various efforts have been taken to integrate herbal medicine as a novel therapeutic agent to prevent and treat kidney injury. The ingredients of green coffee and/or its *in vivo* metabolites were believed to improve kidney damage and its raised biomarkers, due to its antioxidant and anti-inflammatory effect [24, 25].

The health benefits of green coffee beans are due to the presence of physiologically active ingredients such as caffeine, trigonelline, chlorogenic acids, and diterpenes. Specifically, CGAs, abundant bioactive compounds in the green coffee beans, have a health-giving property through direct antioxidant activity, anti-inflammatory activity, and modulatory effects in cells by selectively acting on multiple cell signaling pathways [25]. Recent epidemiological and experimental results indicate that drinking coffee regularly helps to prevent several chronic diseases, such as cardiovascular disease, diabetes mellitus, cancer, Parkinson's disease, and inflammatory conditions [26].

Although the relationship between the consumption of coffee and the risk of renal damage cannot be adequately studied, several studies have shown that coffee consumption has anti-inflammatory and antioxidant activity; hence, it may protect against kidney damage that could be caused by inflammation, apoptosis, and oxidation stress [27, 28]. However, such studies and reports are lacking in our country on the renoprotective effects of green coffee beans, especially on our local species on drug-induced nephrotoxicity disease models.

Therefore, the current study was planned to investigate the renoprotective effects of our locally grown green coffee beans against cisplatin-induced nephrotoxicity in Swiss albino mice.

## 2. Materials and Methods

### 2.1. Study Design

The posttest only control group design was employed.

### 2.2. Ethical Consideration

The ethical clearance was obtained from Jimma University Institutional Review Board (IRB) by approval letter with Ref. No. IHRPGD/718/2020 issued on 24/07/2020. All animal handling procedures and experimental activities were performed in strict accordance with a recommendation from the declaration of nationally and internationally conventional standards for the employment of experimental animals and code of ethics of animal experiments, which comply with scientific and ethical guidelines.

### 2.3. Reagents and Chemicals

Ethanol, methanol, distilled water, renal function test reagents, 10% formalin, paraffin wax, xylene solution, ketamine, H&E stain, cisplatin were from TherDose Pharma Private limited. All chemicals and reagents of analytical grade were used in this experiment.

### 2.4. Acute Oral Toxicity Test

The acute oral toxicity (limit) test was conducted on female rats. Test doses of green coffee beans were calculated in relation to the body weight of every fasted rat and administered via oral gavage at 2000 mg/kg body weight/day [29]. The rats were frequently observed for behavioral changes and common toxicity signs after dosing for the first 24 hours with special consideration being given during the first 4 hours. Thereafter, observation was continued daily for the whole 14 days. No sign of toxicity or mortality was observed within 24 h and over two weeks, respectively.

### 2.5. Collection and Identification of Plant Materials

The ripe green coffee beans (400 g) were collected from farming located near Jimma town, Ethiopia. The plant was authenticated by a botanist in Addis Ababa University as *Coffea arabica* L. and a voucher specimen BL001 was given and deposited at the National Herbarium, College of Natural Sciences, Addis Ababa University.

### 2.6. Preparation of Green Coffee Bean Extract

Extraction of green coffee beans was performed according to the methods [30] with some modifications. The green coffee beans were crushed using the mechanical grinder and followed by maceration in 80% methanol (V/V) for three days with mechanical shaking twice a day. Then, the extract was filtered using Whatman filter paper 1.0 and dried by evaporation using a rotary evaporator. Finally, the filtrate was taken to the freeze dryer to get solid consistency. The final extract was kept in a refrigerator at 4°C, until further use.

### 2.7. Experimental Animals

A total of 30 male Swiss albino mice aged 8–10 weeks (weighing 34–38 grams) were obtained from the experimental animal care unit of the Jimma University, Tropical and Infectious Diseases Research Center. The experimental mice were situated in a plastic cage with stainless steel cover and were kept in the animal laboratory with optimum temperature (22 ± 2°C), optimum ventilation, and a 12-hour light-dark cycle. The cages were kept clean throughout the experiment. Until the initiation of the experiment, all mice were left one week for acclimatization on laboratory conditions. All mice were provided with free access to pellet and water *ad libitum* throughout the experiment. Female mice were excluded from the study because of their cyclic hormonal variations that can affect the biochemical levels.

### 2.8. Preparation of Green Coffee Beans Solution and Dosage Calculation

Per the criteria set out in OECD Guideline 423, LD50 of green coffee extracts was tested for toxicity on mice starting at 2000 mg/kg and found to be greater than 2000 mg/kg. This category includes substances characterized to comparatively low acute toxicity risk with oral LD50 between 2000 and 5000 mg/kg [31]. Based on this, 100 mg/kg/day of green coffee bean extract was taken as a single dose of green coffee bean extract. Likewise, 200 mg/kg/day and 300 mg/kg/day of green coffee bean extract were taken as double and triple doses of green coffee bean extract. The green coffee bean extract was prepared in distilled water of 2 mL/100 g body weight of mice and was administered by oral gavage.

### 2.9. Experimental Protocol

The mice were randomly assigned into five groups with six mice in each group. Each mouse in the given group was identified by giving a number on its tail by permanent marker. The mice were grouped and treated as follows:

  Group I (normal control group): received distilled water for 14 days
  Group II (negative control group): received distilled water for 14 days
  Group III (treatment group): received 100 mg/kg BW/day of green coffee bean extract for 14 days
  Group IV (treatment group): received 200 mg/kg BW/day of green coffee bean extract for 14 days
  Group V (treatment group): received 300 mg/kg BW/day of green coffee bean extract for 14 days

On the first day of the experiment, nephrotoxicity was induced in mice of groups II-V with only one dose of freshly prepared 7.5 mg/kg body weight cisplatin intraperitoneally 1 hour after the administration of green coffee bean extract. The dose was selected based on its efficacy in causing nephrotoxicity in mice. Cisplatin of 1 mg/mL concentration was further diluted in 0.9% normal saline at concentration of 0.5 mg/mL [6]. The cisplatin (TherDose Pharma Private limited) was obtained from the Oncology Pharmacy of Jimma Medical Center.

### 2.10. Measurement of Body Weight and Relative Kidney Weight

The body weight of the experimental mice was measured using triple beam balance at the beginning of the experiment, in the middle of the week, and immediately before being euthanized. The body weight was documented using the corresponding code of each mouse in the group. For the statistical test, the initial and final body weights were taken into account and expressed as mean ± SD. The relative kidney weight was determined for all experimental mice as a percentage of kidneys weight to the final body weight of the mice.

### 2.11. Biochemical Analysis

At the completion of the experiment, the mice were fasted overnight and submitted to euthanasia being previously anesthetized with 100 mg/kg ketamine/12.5 mg/kg xylazine. The blood sample was collected by cardiac puncture and transferred to serum separator tube and centrifuged at 3000 rpm for 10 minutes. Serum creatinine and serum BUN levels were evaluated to determine the kidney function status of experimental mice by using a fully automated chemistry analyzer (ABX Pentra 400, China).

### 2.12. Histopathological Study of Mice's Kidney

The left kidney of each mouse was excised and fixed into formalin of 10% for histopathological examination. After 24 hours of fixation, kidney tissues were dehydrated using ethanol at different concentrations starting from 70% to 100% and xylene solution was used to remove ethanol from tissue and replace this ethanol with fluid that is readily miscible with paraffin wax which enhances the tissue to embed easily with the wax to form tissue blocks. Next, the tissue block was sectioned by a microtome. Then, the staining of the dried section was done with a hematoxylin-eosin stain after removal of paraffin wax using ethanol with a descending concentration and was examined by a light microscope (Olympus X21FS1, Philippines) at 40x magnifications. After examination, photos were taken by a microscope camera (camera KRUSS Optronic, Germany, 3.0 MP, USB 2.0), and the pictures were read and interpreted by a pathologist.

### 2.13. Statistical Analysis

One-way analysis of variance was done to determine statistical differences among all groups of the study. Pairwise comparison was conducted by Tukey post hoc multiple comparison tests. The results of the data were presented as mean ± standard error (SE). The *p* values < 0.05 were considered statistically significant. All statistical analysis was done using SPSS version 25.

## 3. Result

### 3.1. Effect of Green Coffee Beans on Body Weight of the Mice

**Figure 1:**
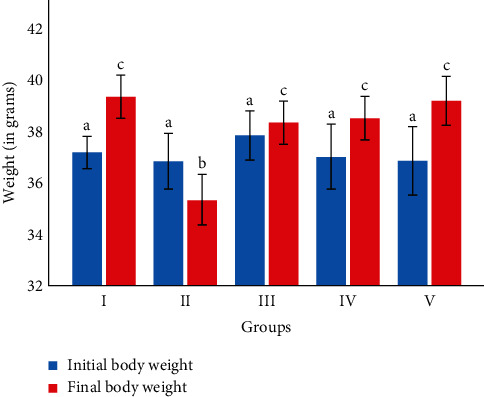
Initial and final body weight of the mice (in grams). The values were expressed as mean ± SD. The sample size is 6 for each group. I: normal control group; II: negative control group; III: received 100 mg/kg/day of green coffee beans; IV: received 200 mg/kg/day of green coffee beans; and V: received 300 mg/kg/day of green coffee beans, respectively. Values with different letters within the same color are statistically significantly different at *p* < 0.05 as tested by Tukey post hoc multiple comparisons.

At the beginning of the experiment, the body weight of experimental mice did not show a significant difference among groups (*p*=0.669) (Figure 1). However, at the end of the experiment, the body weight of experimental mice treated with 100, 200, and 300 mg/kg BW/day of green coffee beans significantly increased compared to the negative control group (*p*=0.047,  0.025,  0.001), respectively (Figure 1). Statistically significant differences in body weight were not found between mice treated with any dose of green coffee beans and the normal control group (*p* > 0.05) (Figure 1). However, the final body weight of cisplatin-induced experimental mice showed significant lowering when compared to the normal control group (*p*=0.001) (Figure 1).

### 3.2. Effect of Green Coffee Beans on Kidney Function Tests

As shown in Table 1, treating the mice with 100, 200, and 300 mg/kg BW/day of green coffee beans significantly decreased serum creatinine (*p*=0.04,  =0.035,   ≤ 0.001) and serum BUN (*p*=0.03,  0.025,   ≤ 0.001) levels compared to the negative control group, respectively. On the other hand, treating mice with any dose of green coffee beans was not found to bring significant changes in serum creatinine and serum BUN levels as compared to the normal control group (*p* > 0.05) (Table 1). However, serum creatinine and serum BUN levels were significantly increased in the negative control group when compared to the normal control group (*p*=0.001,  0.006), respectively (Table 1).

### 3.3. Effect of Green Coffee Beans on Relative Kidney Weight of the Mice

**Figure 2:**
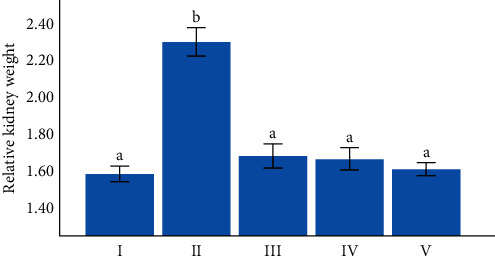
Relative kidney weight of the mice (in percentage). The values were expressed as mean ± SE. The sample size is 6 for each group. I: normal control group; II: negative control group; III: received 100 mg/kg/day of green coffee beans; IV: received 200 mg/kg/day of green coffee beans; and V: received 300 mg/kg/day of green coffee beans, respectively. Values with different letters are statistically significantly different at *p* < 0.05 as tested by Tukey post hoc multiple comparisons.

At the end of the experiment both the right and left kidneys were collected to calculate the relative kidney weight. The relative kidney weight of experimental mice treated with 100, 200, and 300 mg/kg BW/day of green coffee beans significantly reduced compared to the negative control group (*p*=0.03,  =0.027,   ≤ 0.001), respectively (Figure 2). On the other hand, there were no statistically significant differences in relative kidney weight between mice treated with any dose of green coffee beans and the normal control group (*p* > 0.05) (Figure 2). However, the relative kidney weight of the negative control group showed a significant increase when compared to the normal control group (*p* ≤ 0.001) (Figure 2).

### 3.4. Effect of Green Coffee Beans on Histopathological Changes of Kidney

**Figure 3:**
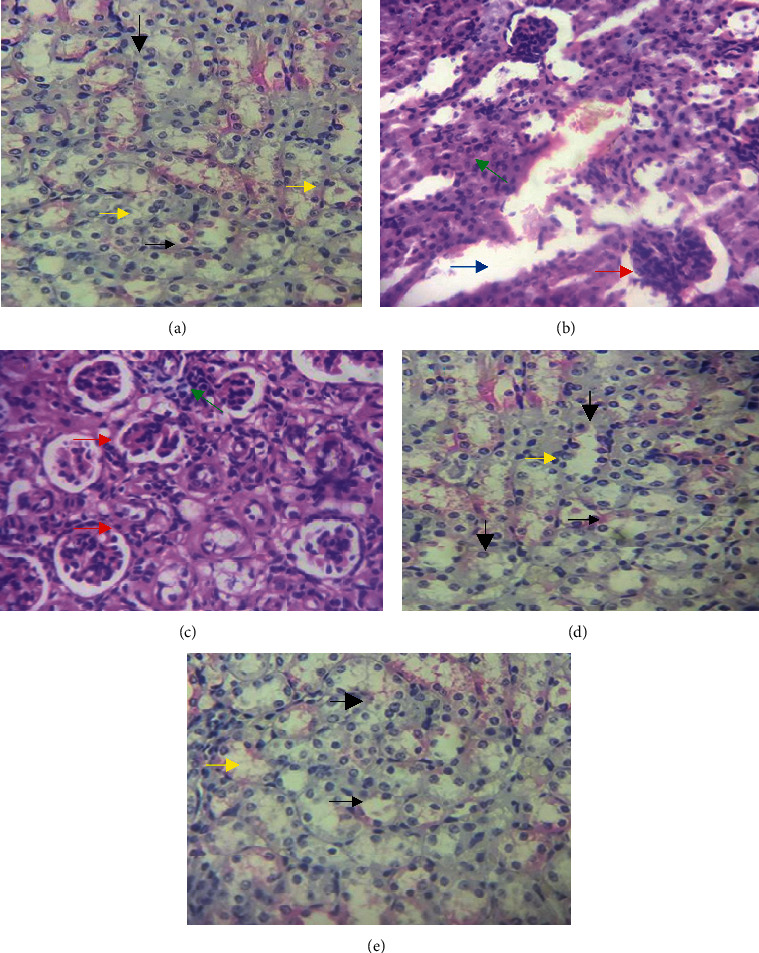
(a) Photomicrograph of kidney of experimental animals (stained with hematoxylin and eosin), where normal proximal tubule (thick arrow), normal distal tubule (thin arrow), and normal glomerulus (yellow arrow) are shown. (b) Photomicrograph of kidney of experimental animals (stained with hematoxylin and eosin), where fibrosis (blue arrow), necrosis (red arrow), and inflammatory infiltration (green arrow) are shown. (c) Photomicrograph of kidney of experimental animals (stained with hematoxylin and eosin), where necrosis (red arrow) and inflammatory infiltration (green arrow) are shown. (d) Photomicrograph of kidney of experimental animals (stained with hematoxylin and eosin), where normal proximal tubule (thick arrow), normal distal tubule (thin arrow), and normal glomerulus (yellow arrow) are shown. (e) Photomicrograph of kidney of experimental animals (stained with hematoxylin and eosin), where normal proximal tubule (thick arrow), normal distal tubule (thin arrow), and normal glomerulus (yellow arrow) are shown.

At the end of the experiment, left kidneys were examined for histopathological changes to assess the protective effect of green coffee bean extract against cisplatin-induced renal damage. Light microscopy examination of renal tissues of mice in the normal control group showed normal glomerulus structure and renal tubular interstitial with no evidence of cell necrosis and inflammatory infiltration (Figure 3(a)). However, histopathological examination of the negative control group that was challenged with cisplatin showed typical renal tissues damage characteristics, for example, necrosis and fibrosis of renal tubular cells and inflammatory infiltration (Figure 3(b)). The mice that received 100 mg/kg BW/day of green coffee beans showed significant improvement in the number of apoptotic and infiltration of inflammatory cells in the kidney (Figure 3(c)). Interestingly, treating the mice with 200 and 300 mg/kg BW/day of green coffee bean extract is evidenced by the normal structure of the kidney (Figures 3(d) and 3(e)).

## 4. Discussion and Conclusion

About 25% of commonly used drugs are potentially nephrotoxic and cisplatin is categorized under one of the nephrotoxic drugs. Cisplatin causes acute kidney damage by induction of oxidative stress, tubule-interstitial inflammation, and apoptosis/necrosis of renal tubular cells [32]. Cisplatin induces metabolic disorders including elevated serum creatinine and blood urea nitrogen as a result of depleted antioxidant status that can lead to pathophysiologic development of nephrotoxicity [33]. Herbal plants such as green coffee beans are considered a natural source of antioxidants because of their phenolic compounds. These compounds have an inhibiting effect on lipid peroxidation and a reducing effect on inflammatory cytokines, hence having a modulating effect on oxidative stress and inflammation [34].

Our results showed a significantly increased body weight gain in mice treated with 100, 200, and 300 mg/kg/day of green coffee bean extract at the end of the experiment as compared with the negative control group. This might be associated with kahweol in green coffee beans that significantly decreases plasma levels of TNF-*α* and IL-6 and hence improves cisplatin-induced appetite loss [35]. However, in contrast to the normal control group, the negative control group showed statistically significant body weight loss. Harmonious with this finding, Lin et al. and Shinsyu et al. reported that male Wistar mice injected on cisplatin showed significantly lessened body weight gain than the normal control group. The possible mechanism by which cisplatin decreased body weight gain in the current study might be through increased production of proinflammatory cytokines which in turn affect neuroendocrine control of appetite leading to anorexia and increased catabolism and induction of gastric stasis [36, 37].

In the current study, the right and left kidneys were collected from all mice and weighed to determine relative kidney weight as percent of final body weight for all groups. As an effect, mice treated with 100, 200, and 300 mg/kg BW/day of green coffee bean extract showed significantly reduced relative kidney weight as compared with the negative control group. This is might be due to the presence of caffeine in green coffee beans that strongly downregulate TGF-*β*, a major mediator of hypertrophic cellular changes, in connective tissues of organs [38]. On the other hand, the obtained result showed statistically enlarged relative kidney weight in the negative control group when compared with the normal control group. In agreement with the finding, Ramkumar et al. reported that 212 male albino mice with cisplatin-induced acute renal failure were evidenced with larger relative kidney weight. Cisplatin injection causes a shift in relative kidney weight in animal subjects. The main mechanism could be due to an increase in glomerular volume and adaptive growth of kidney tissue to restore the lost renal function [39].

The obtained results showed that serum creatinine and serum BUN levels were significantly lower in mice treated with 100, 200, and 300 mg/kg/day of green coffee bean extract when compared to the negative control group. In line with this finding, Amin et al. reported that rats received oral green coffee bean for seven days with the induction of acute renal failure (ARF) showed significantly lower serum creatinine and serum BUN [24]. Comparable studies by Mansour et al. reported that green coffee beans had significantly reduced serum creatinine and serum BUN in cisplatin-challenged mice [40]. These declines in serum creatinine and serum BUN levels in our study may be due to several factors. Kahweol, a natural diterpene in green coffee beans, suppresses oxidative stress and prevents apoptosis of dopaminergic neurons [35]. Above and beyond, the decline in serum creatinine and serum BUN levels might be correlated to chlorogenic acids, the abundant polyphenol in green coffee beans, which shows anti-inflammatory activity and is potent antioxidant against lipid peroxidation [41]. The other mechanism is probably by the antioxidant activity of caffeine that might be responsible for improving kidney function through blocking OCT2 transporter [42].

Serum creatinine and serum BUN levels were significantly higher in the negative control group than in the normal control group. These were in harmony with a previous study reported by Fatima et al. that cisplatin injection could increase serum creatinine and serum BUN by inducing inflammatory processes [43]. A similar finding was also reported by Miller et al. in which injection of cisplatin to mice produced an elevation of renal biomarkers via direct renal proximal tubular cells toxic injury [44]. The serum creatinine and BUN levels did not show a statistically significant difference between the normal control group and groups treated with 100, 200, and 300 mg/kg/day of green coffee bean extract.

Moreover, a kidney histopathology examination was performed to evaluate the ameliorative effects of green coffee beans against kidney injury. Significant improvement was seen in pathological changes of kidney tissues in groups of mice treated with 200 and 300 mg/kg BW/day of green coffee bean extract evidenced by the normal structure of the kidney and this effect is more obvious in Figures 3(d) and 3(e). However, treating mice with 100 mg/kg BW/day of green coffee bean extract showed mild focal lymphocytic infiltrations in the renal parenchyma. This finding is in agreement with Kim et al.'s study, which reported that pathological abnormalities of the kidney showed improvement in mice treated with extract of kahweol. This might be associated with kahweol which reduces proinflammatory cytokines and inhibits signaling pathway that plays important role in the synthesis of proinflammatory cytokines and hence improves the pathological damage of kidney tissues [35]. An additional possible mechanism is linked to the antioxidant bioactive molecules in green coffee beans such as chlorogenic acid and caffeine that inhibit oxidative stress, hence, attenuating histopathological damage [45]. The negative control group showed severe pathological changes of kidney evidenced by diffuse lymphocytic infiltration and this is agreed with Alhoshani et al.'s study on male Wistar rats presenting with inflammatory cell infiltration and tubular fibrosis of the kidney. The severe damage of kidney histopathology found in our study may be due to cisplatin upregulating proinflammatory cytokines together with infiltration of macrophages and neutrophils in kidney tissues which in turn trigger inflammatory cascade [46]. The antioxidant test could not be included in this study. Much research is needed in the future to assess the antioxidant efficacy of green coffees.

In summary, the green coffee bean extract administration for 14 days may contribute to improving kidney injury resulting from cisplatin administration. The green coffee bean extract proved its ameliorative effect on kidney functions and kidney histopathology. Besides, it has better effects on body weight gain and relative kidney weight in a dose-dependent manner with 300 mg/kg green coffee bean extract being the most effective. Therefore, the overall findings of the present study indicate that our locally grown green coffee bean consumption can help to improve nephrotoxicity and its associated complications, such as inflammation, AKI, and CKD.

## Figures and Tables

**Table 1:** Serum creatinine and BUN levels of mice.

Variables (mg/dl)	Groups					*p* value
	I	II	III	IV	V	
Serum creatinine	0.64 ± 0.05	0.99 ± 0.11^abcd^	0.74 ± 0.06	0.71 ± 0.08	0.68 ± 0.07	≤0.001^*∗*^
Serum BUN	39.8 ± 1.3	56.7 ± 6.8^abcd^	44.1 ± 4.8	42.1 ± 3.5	41.6 ± 2.5	≤0.001^*∗*^

The values were expressed as mean ± SE. The sample size is 6 for each group. I: normal control group; II: negative control group; III: received 100 mg/kg/day green coffee beans; IV: received 200 mg/kg/day of green coffee beans; and V: received 300 mg/kg/day of green coffee beans, respectively. ^*∗*^ indicates significant differences among all groups at *p* < 0.05 as tested by one-way ANOVA. Superscript letters (a, b, c, and d) indicate significant differences compared to groups I, III, IV, and V, respectively at *p* < 0.05 as tested by Tukey post hoc multiple comparisons.

## Data Availability

All the data are well verified and authentic and available on request; please contact the corresponding author via lgbati20@gmail.com.

## Abbreviations

AKI: Acute kidney injury
BUN: Blood urea nitrogen
CGA: Chlorogenic acid
CKD: Chronic kidney disease
CVD: Cardiovascular disease
ESKD: End-stage kidney disease
IL-6: Interleukin-6
OCT2: Organic cation transporter 2
TGF-*β*: Transforming growth factor-beta
TNF-*α*: Tumor necrosis factor-alpha.
